# Inhibition of Advanced Glycation End-Product Formation by *Origanum majorana* L. *In Vitro* and in Streptozotocin-Induced Diabetic Rats

**DOI:** 10.1155/2012/598638

**Published:** 2012-09-12

**Authors:** Rosa Martha Perez Gutierrez

**Affiliations:** Laboratorio de Investigación de Productos Naturales, Escuela Superior de Ingenieria Quimica e Industrias extractivas IPN, Avenida Instituto Politécnico Nacional S/N, Unidad Profesional Adolfo Lopez Mateos, cp 07708 México, DF, Mexico

## Abstract

The development of AGE inhibitors is considered to have therapeutic potential in patients with diabetes diseases. The aim of the present study was investigate the effect of methanolic extract of the leaves of *Origanum majorana* (OM) used as spice in many countries on AGEs formation. *In vitro* studies indicated a significant inhibitory effects on the formation of AGEs. Their antiglycation activities were not only brought about by their antioxidant activities but also related to their trapping abilities of reactive carbonyl species such as methylglyoxal, an intermediate reactive carbonyl of AGE formation. The results demonstrate that OM have significant effects on *in vitro* AGE formation, and the glycation inhibitory activity was more effectively than those obtained using as standard antiglycation agent aminoguanidine. OM is a potent agent for protecting LDL against oxidation and glycation. Treatment of streptozotocin-diabetic mice with OM and glibenclamide for 28 days had beneficial effects on renal metabolic abnormalities including glucose level and AGEs formation. Diabetic mice showed increase in tail tendon collagen, glycated collagen linked fluorescence and reduction in pepsin digestion. Treatment with OM improved these parameters when compared to diabetic control and glibenclamide.

## 1. Introduction 

Advanced glycation end-products (AGEs) are the final products of the nonenzymatic reaction between reducing sugars and amino groups in proteins, lipoproteins, and nucleic acids. They are a group of complex and heterogeneous compounds that are known as brown and fluorescent cross-linking substances such as pentoside, nonfluorescent cross-linking products like methylglyoxal-lysine dimers, or nonfluorescent, noncross-linking adducts such as carboxymethyllysine and pyrraline, a pyrrole aldehyde [1]. Recently, AGEs accumulation *in vivo* has been considered to play a major role in the pathogenic process of diabetes and its complications, including neuropathy, nephropathy, retinopathy, and cataract [2] and in other health disorder such as atherosclerosis [3], Alzheimer's disease [4], and normal aging [5]. Thus, the discovery and investigation of compounds with an AGEs inhibitor activity, would certainly offer a potential therapeutic approach for the prevention of diabetes or other pathogenic complications.

*Origanum majorana *L. (majorana) is a herbaceous and perennial plant native to southern Europe and the Mediterranean. For food uses, majorana is employed to flavor sausages, meats, salads, and soups. Culinary herbs have been grown and used for hundreds of years, and they are becoming increasingly popular for their ability to enhance and complement the flavors of a wide variety of foods. Traditionally, it is used as a folk remedy against asthma, indigestion, headache, and rheumatism. Among the herbs of the Lamiaceae family, rosemary has been more extensively studied and its extracts are the first marketed natural antioxidants. Majorana which belongs to the same family, has gained the interest of many research groups as a potent antioxidant [6–9]. 

Phytochemical studies indicate that majorana plant contains polyphenols. Beside these polyphenols, arbutin, 6-O-4-hydroxybenzoyl arbutin, and 2-hydroxy-3-(3,4-dihydroxyphenyl) propionic acid were isolated as moderate antioxidants [10]. The contents of carnosic acid, ursolic acid, and carnosol antioxidant compounds were determined by the HPLC method [11]. In another study, a methanol extract from leaves strongly inhibited rat intestinal *α*-glucosidase. 6-hydroxyapigenin, scutellarein, 6-hydroxyapigenin-7-O-*β*-d-glucopyranoside, 6-hydroxy luteolin-7-O-*β*-d-glucopyranoside, 6-hydroxyapigenin-7-O-(6-O-feruloyl)-*β*-d-glucopyranoside, and 6-hydroxylutcolin-7-O-(6-O-feruloyl)-*β*-d-glucopyranoside were isolated as active principles and related compounds [12]. However, little is known about the biologically active compounds of majorana as a medicinal plant. In this study, the aim is to investigate the AGEs inhibition capacity of extracts from the leaves of *Origanum majorana in vitro *and *in vivo *assays.

## 2. Materials and Methods

### 2.1. Plant Material and Preparation of Extracts

Fresh plants of *Origanum majorana *were collected in the Mexican province of Estado de México. A voucher specimen (no. 7918) was deposited in the Herbarium of the UAM-Xochimilco, for further reference. A total of 300 g of the aerial parts of *O. majorana *were dried and powdered in a mechanical grinder. The grinded material was extracted with 900 mL of hexane, chloroform, and methanol consecutively using a soxhlet apparatus. These extracts were filtered and concentrated by a rotary vacuum evaporator and kept in a vacuum dessicator for complete removal of solvent. An aqueous suspension was prepared using2% (v/v) Tween-80 and then used for oral administration.

### 2.2. *In Vitro* Glycation of Proteins 

#### 2.2.1. Bovine Serum Albumin (BSA)-Glucose Assay

The methodology was based on that of Brownlee et al. [13]. BSA (l0 mg/mL) was incubated with glucose (500 mM) in phosphate buffered-saline (PBS) (5 mL total volume, pH 7.4) and extract containing 0.02% sodium azide at 37°C with a final concentrations of BSA (2 mg/mL), glucose (40 mM), sample (0.1 to 0.5 mg/mL). All the reagent and samples were sterilized by filtration through 0.2 *μ*m membrane filters. The protein, the sugar and the prospective inhibitor were included in the mixture simultaneously. Aminoguanidine was used as an inhibitor positive control. Reactions without any inhibitor were also setup. Each solution was kept in the dark in a capped tube. After 15 days of incubation, fluorescence intensity (excitation wavelength of 370 nm and emission wave-length of 440 nm) was measured for the test solutions. Percent inhibition was calculated as follows:

(1) $$\begin{array}{c} \text{Inhibition}\;\%=\;\left[1-\frac{\left({\text{A}}_{\text{s}}-{\text{A}}_{\text{b}}\right)}{\left({\text{A}}_{\text{c}}-{\text{A}}_{\text{b}}\right)}\right]\times 100, \end{array}$$

where A_s_ = fluorescence of the incubated mixture with sample, A_c_, A_b_ = are the fluorescence of the incubated mixture without sample as a positive control and the fluorescence of incubated mixture without sample as a blank control.

#### 2.2.2. BSA-Methylglyoxal Assay

This assay was modified based on a published method [14]. The assay evaluates the middle stage of protein glycation. BSA and methylglyoxal were dissolved in phosphate buffer (100 mM, pH 7.4) to a concentration of 20 mg/mL and 60 mM, respectively. Extract or fractions were dissolved in the same phosphate buffer. One milliliter of the BSA solution was mixed with 1 mL of methylglyoxal solution and 1 of mL OM extract. The mixture was incubated at 37°C. Sodium azide (0.2 g/L) was used as an aseptic agent. Phosphate buffer was used as a blank. Aminoguanidine and phloroglucinol were used as positive controls. After seven days of incubation, fluorescence of the samples was measured using an excitation of 340 nm and an emission of 420 nm, respectively.

(2)   The  %  inhibition  of  AGE  formation =[1−(fluorescence  of  the  test  groupfluorescence  of  the  control  group)]×100%.

#### 2.2.3. Amadori Activity

 Amadori activity was determined using an after Amadori screening assay [15]. Lysozyme (10 mg/mL) was incubated with 0.5 M ribose in 0.1 M sodium phosphate buffer containing 3 mM sodium azide, pH 7.4 at 37°C for 24 h. Unbound ribose was removed by dialysis against 4 1 of 0.1 M sodium phosphate buffer, pH 7.4 at 4°C for 48 h with 5-6 changes. Following dialysis, the protein concentration was determined using the Bio-Rad standard protein assay kit based on the Bradford dye-binding procedure [16]. Dialysed lysozyme (10 mg/mL) was reincubated with 10 mg/mL of OM and aminoguanidine in 0.1 M sodium phosphate buffer containing 3 mM sodium azide, pH 7.4 at 37°C for 15 days. 

#### 2.2.4. Glycation of Hemoglobin

Glassware was previously sterilized. The experiment was performed on a specially treated bench to avoid any possible contamination. Glucose (2 g/dL), hemoglobin (12 g/dL), extract (5 g/dL), and glutathione (200 mM) were dissolved in distilled-sterilized water. This hemoglobin solution was diluted with three times as much water for the negative control group. Hemoglobin, glucose, and water were mixed at a ratio of 1 : 1 : 2 (v/v/v). Isolated or glutathione was added instead of water to the positive control group. These mixtures were incubated for 5 days at 37°C with continuous stirring (70 rpm). The amount of glycated hemoglobin (% GHb) was determined using an ion capture component set (IMx system, Abbott laboratories, USA). The amount of hemoglobin A_lc_ (% HbA_lc_) was calculated by defining the equation used to convert IMx glycated hemoglobin (% GHb) to standardized percent of hemoglobin A_lc_ (% HbA_lc_). 

#### 2.2.5. LDL Oxidation Measurement

Amphotericin B was dissolved in methanol first and then added to an LDL solution, with or without compound treatment, for final concentration of 5 and 10 *μ*M de Amphotericin B. One milliliter of CuS0_4_ (10 *μ*M) was used to initiate LDL oxidation in 10 mL of an LDL solution sample. After incubating the LDL solution at 37°C for 72 h, the method of Jain and Palmer [17] was used to measure malondialdehyde (MDA) formation (nmol/mg LDL protein). Briefly, 0.2 mL LDL solution was suspended in 0.8 mL PBS. Then 0.5 mL trichloroacetic acid (TCA; 30%) was added. After vortexing and standing in ice for 2 h, samples were centrifuged at 1500 ×g for 15 min. Supernatant (1 mL.) was mixed with 0.25 mL, thiobarbituric acid (TBA) (1%), and the mixture was heated in a boiling water bath for 15 min. The absorption of MDA-TBA complex was measured at 532 nm. The formation of conjugated diene (CD), a lipid oxidation product, in LDL also was determined according to the method described by Esterbauer et al. [18]. The lipid oxidation of an LDL solution containing 5 or 10 *μ*M of each compound was initiated at 37°C by 0.1 mM CuC1_2_. Absorbance at 234 nm was continuously recorded for 60 min at 37°C by a Hitachi U-2001 spectrometer with a constant temperature recirculator. The lag phase, expressed in minutes, was defined as the period where no oxidation occurred. A longer lag phase indicated less CD formation. 

#### 2.2.6. *In Vitro* Glycation of LDL

LDL glycation was performed according to the method described in Li et al. [19]. Briefly, 50 mM glucose in PBS (pH 7.4) was added to an LDL solution (0.3 mg protein/mL) with and without compound treatment. Sodium azide at 0.02% was used as antibiotic to prevent bacterial growth. This solution was sterile filtered, covered with N_2_, and stored for 6 d at 37°Cin the dark. After glycation, the solutions were dialyzed against PBS (20 mL, against 4 L) at 4°C for 40 h. Then glycated LDL was separated from nonglycated LDL by applying a GlycoGel II column (Pierce, Rockford, IL, USA), in which 500 *μ*L LDL solution was loaded on the column, and glycated LDL was eluted with 2 mL, sorbitol buffer, pH 10.25. Neither copper nor any other oxidant was used for the experiments on LDL glycation. The method of Duell et al. [20] was used to measure LDL glycation level. LDL solution (200 *μ*L) was mixed with 200 *μ*L 4% NaHCO_3_ and 200 *μ*L 0.1% trinitrobenzoic acid. This mixture was flushed with N_2_, sealed, and incubated at 37°C in the dark. After 2 h, the absorbance at 340 nm was measured spectrophotometrically. The blank was a mixture of LDL and NaHCO_3_ in PBS. LDL glycation is reported as relative reduction in the level of free *ε*-amino groups of L-lysine when compared with LDL solution in the absence of glucose. During LDL glycation, samples were treated with or without EDTA (0.5 mM), and LDL oxidation level was also determined. 

#### 2.2.7. Experimental Animals

Study was conducted in male Wistar rat weighing about 180–200 g. They were procured by the bioterium of the National School of Biological Sciences IPN and were housed in microlon boxes in a controlled environment (temperature 25 ± 2°C) with standard laboratory diet and *ad libitum* water. Animals were acclimatized for a period of three days in their new environment before initiating the experiment. Litter was renewed three times a week to ensure hygiene and maximum comfort of the animals. The experiments reported in this study were following the guidelines stated in Principles of Laboratory Animal Care (NIH publication 85-23, revised 1985 and the Mexican Official Normativity (Norma Official Mexicana) NOM-062-Z00-1999). 

#### 2.2.8. Experimental Design

In experimental rats were kept in wire-bottomed cages, and exposed to a 12-h light/dark cycle. The room temperature and humidity were maintained automatically at about 25°C and 60%, respectively. These animals had *ad libitum *access to commercial food (Purina) and water. After several days of adaptation, the mice were randomly separated into normal control (*n* = 5) and diabetic groups. The diabetic groups were given an intraperitoneal (i.p.) injection of streptozotocin 45 mg/kg body weight in 10 mM citrate buffer (pH 4.5). Animals receiving an injection of citrate buffer were used as a normal control. After 10 days of the streptozotocin or vehicle injection, the blood samples were obtained from the tail vein between 10:00 and 11:00 am. Rat with a blood glucose level higher than 300 mg/dL were used as diabetic rat: and randomly divided into five experimental groups. Diabetic animals were treated orally with the methanol extract dissolved in water at doses of 200 mg/kg/day by oral gavage while their control group was only given water. 28 days later, after renal perfusion through the renal artery with ice-cold physiological saline, the kidneys were removed from each rat. 

#### 2.2.9. Serum Parameters

Glycated protein was measured by the thiobarbituric acid assay of McFarland et al. [21] in which nonenzymatically bound glucose is released as 5-hydroxymethylfurfural and quantitated colorimetrically.

#### 2.2.10. AGE Level in Kidney

The renal AGE level was determined by the method of Nakayama et al. [22]. In brief, minced kidney tissue was dilapidated with chloroform and methanol (2 : 1, v/v) overnight. After washing, the tissue was homogenized in 0.1 N NaOH, followed by centrifugation at 8000 ×g for 15 min at 4°C. The amounts of AGEs in these alkali-soluble samples were determined by measuring the fluorescence at an emission wavelength of 440 nm and an excitation wave length of 370 nm. A native BSA preparation (l mg/mL of 0.1 N NaOH) was used as a standard, and its fluorescence intensity was defined as one unit of fluorescence. The fluorescence values of samples were measured at a protein concentration of 1 mg/mL and expressed in arbitrary units (AU). 

#### 2.2.11. Mitochondrial TBA-Reactive Substance Level in Kidney

Mitochondria were prepared from kidney homogenate by differential centrifugation (800 ×g and 12000 ×g, resp.) at 4°C according to the methods of Jung and Pergande [23], with minor modifications. Each pellet was resuspended in preparation medium and the concentration of TBA-reactive substance was determined by the method of Uchiyama [24]. 

#### 2.2.12. Glycation of Tail Tendon Collagen

Tendons separated from tails of experimental mice were washed thoroughly in saline solution at 4°C. Acid hydrolysis of the tendons was carried out at 121°C for 4 h. Hydroxyproline was estimated in hydrolysed tendon collagen samples according to the method of Woessner [25]. Collagen content in the mouse tail tendon was expressed in mg collagen/100 mg tissue assuming that collagen weighs 7.46 times hydroxyproline. Extent of collagen glycation in tail tendon was assessed by phenol-sulfuric acid method [23] and expressed as, *μ*g of glucose/mg collagen. Clean tendons (50 mg, wet weight) were digested in freshly prepared pepsin solution (1 mg/mL in 0.5 M acetic acid, 5 mL) for 24 h at 37°C to determine the amount of pepsin soluble collagen [26]. Samples of pepsin-digested collagen (0.25 mL) were mixed with 2.75 mL of 200 mM phosphate buffer (pH 7.5). Collagen-linked fluorescence was quantified at 365 nm excitation and 416 nm emission, relative to the standard quinine sulfate solution (1 *μ*g/mL) and expressed as AU/mg collagen. 

#### 2.2.13. Statistical Analysis

Data are presented as means ± S.E.M. The effect of extracts on each parameter was examined using the one-way Analysis of Variance. Individual differences among groups were analyzed by Dunnett's test and significance was accepted at *P* < 0.05.

## 3. Results

### 3.1. *In Vitro* Glycation of Proteins 

#### 3.1.1. BSA-Glucose and BSA-Methylglyoxal Assays

In order to determine the inhibitory effect of the methanol extract from *O. majorana *on AGEs formation, several assay methods have been proposed including assays based on inhibition of specific fluorescence generated during the course of glycation and AGEs formation, and assays based on the inhibition of AGEs-protein cross-linking. OM, phloroglucinol and aminoguanidine exhibited higher inhibitory activity against AGEs formation after incubation at 37°C for 15 days, with an IC_50_ value of 0.310, 0.070, and 0.323 mg/mL, respectively (Table 1). Methylglyoxal-mediated protein glycation inhibition was evaluated for OM which exhibited a substantial activity, compared with phloroglucinol and aminoguanidine (Table 1), with IC_50_ values of 0.190, 0.060 and 0.195 mg/mL, respectively. In addition, effect of HS on the formation of AGEs induced by ribose produces an inhibition of 42.9% compared to aminoguanidine with 58.3% inhibition.

#### 3.1.2. Amadori Activity

Lysozyme generated cross-linked advanced glycation end-products. This test is used to evaluate the ability of the extract to inhibit the cross-linking of lysozyme in the presence of ribose. 5 and 10 mg/mL of extract and 1 mM aminoguanidine were found to inhibit the formation of AGE (fluorescence) after 15 days of incubation, as shown in Table 1 with values of 64.7% and 58.3% for methanolic extract of MO and aminoguanidine, respectively. Methanolic extract of OM has activity and inhibits cross-linked advanced glycation end-products.

#### 3.1.3. Glycation of Hemoglobin

Table 2 shows the amount of glycated hemoglobin (% GHb). When hemoglobin was used alone (NC), the amount of glycated hemoglobin was 9.5%. This noticeably increased with the addition of glucose to a 27.6% (PC). Nonetheless, it decrease significantly with the treatment of OM (18.6%) and dropped further with the treatment of glutathione (8.1%). The amount of hemoglobin A_1c_ (% HbA_1c_), corresponds to a specific subfraction of glycated hemoglobin, it is lower than the amount of glycated hemoglobin. However, it showed a similar tendency in the percentage of glycation. This result indicates that OM have the most potent glycation inhibition at the early stage of protein glycation at a concentration, of 10 mg/mL. These plant therefore can effectively prevent HbA_1c_ formation. 

#### 3.1.4. Serum-Glycosylated Protein

Glycated protein levels in blood serum of diabetic mice were significantly higher than those of control mice as expected. At the end of the experiment after treatment with methanolic extract OM glycosylated protein, in diabetic mice were statistically decreased (Table 2). 

#### 3.1.5. LDL Oxidation Measurement

Amphotericin B treatments at 5 and 10 *μ*M significantly increased LDL oxidation levels as by determined MDA formation. However, the presence of the extract at 5 and 10 *μ*g/mL significantly reduced 10 *μ*M amphotericin B-induced LDL oxidation (Table 3, *P* < 0.05). 

#### 3.1.6. *In Vitro* Glycation of LDL

LDL treated with 50 mM glucose significantly increased glycation level (Table 4, *P* < 0.05). Under EDTA protection, the presence of the OM 5 and 10 *μ*g/mL significantly reduced LDL glycation. On the other hand, both LDL oxidation and LDL glycation significantly increased when LDL was treated with 50 mM glucose without EDTA protection. The presence of OM at 5 and 10 *μ*g/mL significantly reduced both LDL oxidation and glycation when compared with controls (*P* < 0.05), acting as antioxidative and antiglycative agent. 

#### 3.1.7. Renal Weight, AGE, and Mitochondrial TBA-Reactive Substance Levels

Kidney weight, renal AGEs and mitochondrial thiobarbituric acid-reactive substance was very elevated in diabetic mice compared to the control group (Table 5). These levels were reduced to almost in range values by the administration of the different isolated. The mitochondrial thiobarbituric acid-reactive substance was increased to 2.09 nmol/mg protein compared with the 1.81 mmol/mg protein of the control mice. These levels were equally decreased by the administration of OM and of aminoguanidine; additionally, the effect observed in the 200 mg/Kg of OM-treated group was the same as in the aminoguanidine-treated group. The level of glucose in the diabetic control group increased during the period of the experiment and the administration of the extract did not have an effect on it. Symptoms in diabetic animals are increased kidney lipid peroxidation (TBARS), reduction in antioxidant defense and increase in renal AGE. This was in agreement with the present study results that confirmed by Maillard-type fluorescent measurement, renal AGEs accumulation in streptozotocin-induced diabetic mice. For these reasons, we first assessed the effect of isolated on renal AGE accumulation and thiobarbituric acid reactive substance. The fluorescence values of samples were measured at a protein concentration of 1 mg/mL and expressed in AU compared with a native BSA preparation. 

#### 3.1.8. Glycation of Tail Tendon Collagen

Collagen was measured in rat-tail tendon after acid hydrolysis. Diabetic rats showed a significant increase in tail collagen, collagen glycation, and collagen-linked fluorescence and reduction in pepsin-digestible collagen (Table 6). Inter- and intramolecular cross-linking with collagen is formed as a result of glycation which is responsible for resistance to pepsin digestion. Treatment with OM and glibenclamide reversed these parameters with respect to the diabetic control. Treatment with OM significantly reduced the levels of collagen-linked fluorescence, which is in agreement with the *in vitro *BSA glycation study. Solubility pattern was also restored, with a relative increase in pepsin soluble collagen. These changes indicated a reduction in cross-linking of collagen proteins in isolated treated diabetic animals. 

## 4. Discussion

Increased glycation during hyperglycaemia can cause intra- or intermolecular cross-linking of proteins as they accumulate advanced glycation end-products. Numerous studies have shown that build up of cross-linked advanced glycation end-products on long-lived proteins may underlie the development of complications affecting diabetes and ageing. Furthermore, the levels of serum advanced glycation end-products reflect the severity of these complications whereas therapeutic interventions aimed at reducing advanced glycation end-products can inhibit or delay their progression [2]. 

In this study we found that OM inhibited the formation of methylglyoxal derived advanced glycation end-products in a bovine serum-albumin-methylglyoxal system, and may also act by blocking conversion of dicarbonyl intermediates to advanced glycation end-products. Furthermore, ours results show that OM could react with carbonyl groups from reducing sugars, Amadori adducts and dicarbonyl intermediates therefore blocking their conversion to advanced glycation end-products. Dicarbonyl intermediates such as methylglyoxal have received considerable attention as mediators of advanced glycation end-product formation and are known to react with lysine, arginine and cysteine residues in proteins to form glycosylamine protein cross-links [27]. Reincubation of dialyzed lysozyme generated cross-linked advanced glycation end-products that were inhibited in the presence of increasing concentrations of OM and aminoguanidine. Methanolic extract have Amadori activity and inhibit cross-linked advanced glycation end-products at concentrations of 5–10 mg/mL. 

 In the present study, typical characteristics of diabetes were shown. First is the increase of serum glycosylated protein, which is a parameter caused by glucose and other reducing sugars such as ribose and fructose reacting with the amino residues of proteins to form Amadori products, for instance, glycosylated hemoglobin (HbA_1c_) and the O_2_^−^ are also generated in the process of AGE formation. OM could directly decrease the formation of glycated hemoglobin, possibly as a result of its antioxidative activity [6–9].

 The next is abnormal lipid metabolism, which can lead to lipid peroxidation with reactive oxygen species (ROS) and renal lipid accumulation, which plays a role in the pathogenesis of diabetic nephropathy. Nonenzymatic glycation of LDL, is accompanied by oxidative, radical-generating reactions. In the presence of EDTA, oxidation was not responsible for the observed glycation; in this instance, glycation may be due simply to the interaction between LDL protein and glucose. On the other hand, elevated levels of LDL glycation were observed when LDL oxidation was not suppressed. In this condition, the oxidation from LDL should be an important contributor toward the elevated glycation because OM, had a powerful antioxidative and antiglycative agent. That is, OM might first retard the oxidation that occurred on LDL lipids, and then retard the subsequent oxidation-related glycation. Such results bear out that LDL glycation is strongly related to its oxidation [28], and also support the idea that delaying LDL oxidation is helpful in retarding LDL glycation. 

 Several lines of studies have provided substantial evidence that multiple factors caused by hyperglycemia contribute to the development of diabetic kidney disease. Among them, the impacts of AGEs have been recognized over a wide range, resulting in the expression and activation of pathogenic mediators implicated in the development of diabetic nephropathy, such as extracellular matrix, oxidative stress, cytokines, and growth factors, viareceptor-dependent and/or independent pathways. Therefore, we first demonstrated renal AGE accumulation and the mitochondrial lipid peroxidation level. As a result, diabetic control rats showed increased kidney weight and AGE accumulation significantly, indicating renal hypertrophy, and also showed an increased level of TBA-reactive substance. Oral administration of OM ameliorated these changes. Particularly, OM successfully reduced AGE and TBA-reactive substance level at the dose of 200 mg/kg suggesting that *Origanum majorana *suppressed the state of oxidative stress, and decreased the levels of serum protein and hemoglobin glycosylated significantly suggesting that it would inhibit oxidative damage and irreversible renal damage caused by the protein glycation reaction under diabetes.

STZ-induced diabetes characterized by hyperglycemia caused a significant increase in rat tail tendon collagen, glycated collagen, collagen-linked fluorescence, and reduction in pepsin-digested collagen. Excessive collagen can result from an imbalance between its synthesis and degradation by interstitial collagenases. Collagenous proteins are especially exposed to glycation because they contain several lysine, hydroxyl lysine, and arginine residues with free amino groups, have a very slow turnover rate and are exposed to ambient levels of glucose [29]. Glycation of collagen leads to formation of AGE and an increase in collagen-linked fluorescence. Glycation of collagen interferes with the activation of metalloproteinases, the enzymes responsible for collagen degradation [30]. Inter- and intramolecular cross-links with collagen are formed as a result of glycation which are responsible for resistance to pepsin digestion. Treatment with isolated and glibenclamide reversed these parameters with respect to diabetic control. Reduction in collagen may be attributed to the significant decrease in blood glucose and consequent decrease in nonenzymatic glycation and deposition of collagen in diabetic rats treated with isolated and glibenclamide. Treatment with isolated and glibenclamide significantly reduced the levels of collagen-linked fluorescence. OM significantly inhibited accumulation of AGE compounds compared to glibenclamide which is in agreement with *in vitro *BSA glycation study. OM and glibenclamide improved the solubility pattern with a relative increase in pepsin soluble collagen. These changes indicated a reduction in cross-linking of collagen proteins in treated diabetic rats.

## 5. Conclusions

In conclusion, our study showed that *Origanum majorana *was effective in inhibiting the formation of AGEs. The antiglycation activities of *O. majorana *were attributed in part to their antioxidant activity and its abilities to scavenge reactive carbonyls. The ability of OM to react with carbonyls was the major mechanism for protein glycation inhibition. Furthermore, OM alleviated oxidative stress under diabetic conditions through the inhibition of lipid peroxidation, prevent and/or delay the onset renal damage. These results suggested that OM might prevent or improve the AGE associated chronic conditions. Therefore, *O. majorana *could be a candidate for use in studies looking at the effects of natural herbal complement in the prevention of diabetes complications, since it possesses both antioxidant and antyglycation activities.

## Figures and Tables

**Table 1:** The inhibitory effects of methanol extract from *O. majorana *(OM) and aminoguanidine on the formation of advanced glycation end-products (AGEs), *in vitro *induced by glucose, methylglyoxal and ribose.

Inducer	Treatment	AGEs IC_50 _(mg/mL)
Glucose	Methanol extract (OM)	0.310 ± 0.054
	Aminoguanidine	0.323 ± 0.075
	Phloroglucinol	0.070 ± 0.0032

Methylglyoxal	Methanol extract (OM)	0.190 ± 0.028
	Aminoguanidine	0.195 ± 0.015
	Phloroglucinol	0.060 ± 0.0083

Lysozyme/ribose	Methanol extract (OM)	64.7%
	Aminoguanidine	58.3%

Data are mean ± standard deviation of triplicate tests.

**Table 2:** The inhibitory effects of methanol extract of *O. majorana * on glycosylated protein glycated, hemoglobin GHb and HbA_1c_.

Groups	GHb	HbA_1c_	Glycated protein (nmol/mg protein)
Negative control	8.9 ± 0.06	7.9 ± 0.98	15.3 ± 1.47
Positive control	27.6 ± 1.34	17.5 ± 1.56	23.7 ± 2.19
Methanol extract	18.6 ± 1.53^a^	14.9 ± 1.25^a^	19.1 ± 2.04^a^
Glutathione	8.1 ± 0.08^a^	9.0 ± 0.67^a^	—
Aminoguanidine	—	—	20.2 ± 1.87^a^

Negative control: incubation with hemoglobin (30 mg/mL), positive control: incubation with hemoglobin (30 mg/mL) + glucose (0.278 mM), methanol extract: incubation with hemoglobin (30 mg/dL) + glucose (0.278 mM) + methanol extract (10 mg/ mL), glutathione: incubation with hemoglobin (3 mg/dL) + glucose (2.7 mM) + glutathione (0.5 mM). Data as expressed as ± SD; ^a^ *P* < 0.05 versus positive control values.

**Table 3:** The prooxidant effect of amphotericin B (AB) at 5 and 10 *μ*M, and the antioxidant protection of 5 and 10 *μ*g/mL of OM against 10 *μ*M, AB-induced malondialdehyde (MDA) formation (nmol/mg LDL protein) and conjugated diene (CD) formation after a 72-h incubation at 37°C.

Groups	MDA formation (nmol/mg LDL protein)	CD formation lag phase (min)
Control	13.26 ± 2.43	7.8 ± 2.60
AB 5 *μ*M	22.56 ± 5.19^a^	46.7 ± 1.94^a^
AB 10 *μ*M	34.29 ± 2.28^a^	58.3 ± 2.87^a^
AB 10 *μ*M + OM (5 *μ*g/mL)	17.31 ± 3.41^a^	20.6 ± 3.28^a^
AB 10 *μ*M + OM (10 *μ*g/mL)	11.56 ± 5.73^a^	13.1 ± 1.76^a^

Values are expressed as mean ± SD, ^a^significantly (*P* < 0.05) different from control, where the significance was performed by one-way ANOVA followed by *post hoc* Dunnett's test.

**Table 4:** Protective effect of 5 and 10 *μ*g/mL, of OM on LDL against 50 mM glucose-induced glycation and oxidation with or without 0.5 mM EDTA treatment.

Treatment	With EDTA		Without EDTA	
	Glycation	Oxidation	Glycation	Oxidation
LDL	2.8 ± 0.56	3.7 ± 0.84	3.8 ± 0.93	21.03 ± 2.31
LDL + glucose	17.2 ± 3.19^a^	4.3 ± 1.39^a^	22.65 ± 3.28^a^	58.80 ± 3.18^a^
OM (5 *μ*g/ml)	5.4 ± 0.96^a,b^	3.3 ± 0.57^b^	15.14 ± 2.74^a,b^	36.49 ± 4.35^a,b^
OM (10 *μ*g/ml)	4.1 ± 0.74^b^	2.9 ± 0.98^b^	13.86 ± 2.52^a,b^	31.83 ± 2.06^a,b^

Values are expressed as Mean ± SD (*n* = 6), ^a^Significantly (*P* < 0.05) different from LDL group. ^ b^Significantly (*P* < 0.05) different from LDL + glucose, where the significance was performed by oneway ANOVA followed by post hoc Dunnett's test.

**Table 5:** Effect of OM on renal mitochondrial TBA-reactive substance, renal weight and AGE levels.

Groups	TBA-reactive substance (nmol/mg protein)	Renal weight (g)	AGE (AU)
Normoglucemic	1.81 ± 0.031^a^	0.73 ± 0.049^a^	15.98 ± 3.26^a^
Diabetic	2.09 ± 0.012	1.08 ± 0.020	24.25 ± 3.73
OM	1.79 ± 0.065^a^	0.92 ± 0.054^a^	11.48 ± 3.42^a^
Aminoguanidine	1.80 ± 0.037^a^	0.97 ± 0.036^a^	12.87 ± 2.89^a^

Data as expressed as ± SD; ^a^ *P* < 0.05 versus diabetic control values.

**Table 6:** Effect of OM on the glycation of tail tendon collagen.

Treatment	Total collagen (mg/100 mg tendon)	Pepsin digested (mg/100 mg tendon)	Collagen glycation (*μ*g glucose/mg collagen)	Fluorescence (AU/mg collagen)
Normal	37.6 ± 1.57	3.46 ± 0.039	2.98 ± 0.38	2.85 ± 0.051
Diabetic	75.9 ± 1.43	1.47 ± 0.075	14.57 ± 0.17	24.63 ± 0.73
Diabetic + OM	44.3 ± 2.19^ab^	2.54 ± 0.019^ab^	9.32 ± 0.58^ab^	16.80 ± 0.60^ab^
Diabetic + Glibenclamide	47.1 ± 1.56^ab^	2.19 ± 0.026^ab^	8.09 ± 0.52^ab^	17.21 ± 0.84^ab^

Data as expressed as ±SD; *n* = 5; ^a^versus normal control; ^b^versus diabetic control.
